# Effects of cinnamon on anthropometric indices and headache-related disability of patients with migraine: A randomized double-blind placebo-controlled trial 

**DOI:** 10.22038/AJP.2023.22874

**Published:** 2024

**Authors:** Azadeh Zareie, Mohammad Bagherniya, Amirhossein Sahebkar, Manoj Sharma, Fariborz Khorvash, Akbar Hasanzadeh, Gholamreza Askari

**Affiliations:** 1 *Nutrition and* *Food Security Research Center, Isfahan University of Medical Sciences, Isfahan, Iran*; 2 *Department of Community Nutrition, School of Nutrition and Food Science, Isfahan University of Medical Sciences, Isfahan, Iran*; 3 *Anesthesia and Critical Care Research Center, Isfahan University of Medical Sciences, Isfahan, Iran*; 4 *Biotechnology Research Center, Pharmaceutical Technology Institute, Mashhad University of Medical Sciences, Mashhad, Iran*; 5 *Applied Biomedical Research Center, Mashhad University of Medical Sciences, Mashhad, Iran*; 6 *Polish Mother's Memorial Hospital Research Institute (PMMHRI), Lodz, Poland*; 7 *School of Pharmacy, Mashhad University of Medical Sciences, Mashhad, Iran*; 8 *Environmental & Occupational Health, School of Public Health, University of Nevada, Las Vegas, NV, USA*; 9 *Department of Neurology, Isfahan University of Medical Sciences, Isfahan, Iran*; 10 *Departments of Epidemiology and Biostatistics, School of Health, Isfahan University of Medical Sciences, Isfahan, Iran*

**Keywords:** Cinnamon, Migraine, Anthropometry, Headache, Weight

## Abstract

**Objective:**

Increased body mass index (BMI) seems to be a risk factor for migraine attacks. Cinnamon has anti-inflammatory, neuroprotective, and anti-obesity effects. This study aimed to assess the effects of cinnamon on anthropometric indices and headache-related disability of patients with migraine.

**Materials and Methods:**

This study was conducted as a randomized, double-blind, placebo-controlled trial involving 50 migraine patients. Patients were randomized to receive either 600 mg cinnamon powder or placebo capsules for two months. Height, body weight (BW), waist circumference (WC), and hip circumference (HC) were measured.

Furthermore, Minimal or Infrequent Disability (MIDAS) and Headache Daily Result (HDR) Questionnaires were recorded.

**Results:**

At the end of the treatment period, BW and BMI did not change in the intervention group; however, both factors were significantly increased in the placebo group (p=0.001). The change of WC, HDR and MIDAS was significantly different between the intervention and placebo groups (p<0.001). Furthermore, HC and WHR significantly decreased (p=0.001).

**Conclusion:**

Cinnamon seems to have beneficial effects on anthropometric indices and headache disability of migraine patients.

## Introduction

Migraine is one of the most common primary headaches. It is known as the third-highest disabling disorder worldwide (Di Renzo et al., 2018). It is prevalent in approximately 12% of the Western world’s population and 14% of the Iranian population (Farhadi et al., 2016). On the other hand, overweight and obesity are one of the current and increasing health problems with more than 1.9 billion overweight and obese adults all over the world (Schlesinger et al., 2019). Obesity is associated with an increased risk of several chronic diseases such as diabetes, dyslipidemia, hypertension, cardiovascular diseases, cancer, and pain disorders such as headaches (Peterlin et al., 2010). 

A growing body of evidence indicates that overweight and obesity may be associated with a higher risk of migraine. Higher body mass index (BMI) is linked to more severe headaches, and increased frequency, and poorer quality of life among migraine patients (Miri et al., 2018; Pavlovic et al., 2017). Migraine prevention medications can increase the body weight (BW) and BMI (Taylor, 2008), and increased BW and BMI have adverse effects on migraine symptoms; thus, finding a safe, novel and practical strategy to control weight gain to prevent the progression of headaches in patients is necessary. Despite the different weight control strategies, complementary and alternative approaches with comparatively fewer side effects have attracted significant attention. Hence, herbal supplementation might be used as an easier and more practical alternative approach (Mousavi et al., 2020). 

The bark of various cinnamon species as the most important and popular cooking spice has been used worldwide, also it is a traditional medicine. Several potential health benefits of cinnamon have been known including anti-inflammatory, antioxidant, anti-neuroinflammatory, neuroprotective, insulin-sensitizing, and anti-obesity (Rao and Gan, 2014). Cinnamaldehyde, polyphenols, and flavonols are the most important constituents of cinnamon that have antioxidant and anti-obesity properties (Mousavi et al., 2020). 

Previous studies indicate that cinnamon has protective effects against metabolic syndrome's aspects, non-alcoholic fatty liver disease (NAFLD), and obesity (Bagherniya et al., 2018; Gupta Jain et al., 2017; Mollazadeh and Hosseinzadeh, 2016). Therefore, it seems that BW and BMI control with cinnamon consumption can be a preventive approach for migraines. To the authors' knowledge, there has been no prior randomized controlled trial to assess the effects of cinnamon supplementation on changing anthropometry indices and disability in migraine patients. Hence, the main aim of the present study was to evaluate the effect of cinnamon supplementation on anthropometry status and headache disability of migraine patients.

## Materials and Methods

**Sample size**

According to the following formula, (Billings et al., 2017; Wittes, 2002), the sample size of this randomized double-blinded placebo-controlled trial was calculated based on 80% power, an alpha level of 0.05, and a potential dropout rate of 10%. It was calculated that 50 participants (i.e. 25 participants in each group) would be needed to detect 20% differences between the groups.

$n-\frac{{\left(Z_{1}+Z_{2}\right)}^{2}(2S^{2})}{d}$

**Participants**

A total of 50 eligible participants who visited one expert neurologist at Khorshid and Imam Mousa Sadr Clinics, Isfahan University of Medical Sciences, Isfahan, Iran were recruited from September 2018 to November 2018. The migraine condition was confirmed by a single expert neurologist based on the third edition of the International Classification of Headache Disorders (ICHD-3) (Galioto et al., 2018). 

Inclusion criteria were as follows: 1) aged 20-50 years old; 2) migraine without aura that was diagnosed by one expert neurologist; 3) willingness to participate in the study after filling the written informed consent; and 4) having normal to moderate anxiety, stress, and depression status according to the DASS-21 questionnaire (Henry and Crawford, 2005). 

Patients were excluded if they had one these criteria: 1) patients with tension-type headache; 2) having chronic diseases such as chronic kidney or gastrointestinal diseases; 3) patients with a history of cinnamon sensitivity and allergy; 4) taking any antioxidants, cinnamon supplement, or anti-inflammatory drugs; 5) being menopause, pregnant or breastfeeding; or 6) taking any weight loss medication and having a weight loss diet. 

**Study design and intervention**

This study is part of the protocol that was designed as a double-blinded placebo-controlled randomized trial previously described in detail elsewhere (Zareie et al., 2020). The whole protocol was approved by the Ethics Committee of Isfahan University of Medical Sciences, (ethics code: IR.MUI.RESEARCH.REC.1397.185). The trial has also been registered in the Iranian Center for Clinical Trials (No. IRCT20121216011763N36). Before entering the study, all participants were informed of the study protocol and completed the written informed consent. This study and manuscript also adhered to the CONSORT guidelines.

All participants were randomized into intervention and placebo groups. Permuted four-blocked randomization method was used for randomization. The Creating a random sequence was conducted by an independent well-trained nutritionist and then the process was kept in opaque, sealed, numbered envelopes until the end of the eligibility criteria evaluation. Investigators and participants were blinded to the randomization codes until the completion of the final analyses. 

Patients in each group received one cinnamon or placebo capsule after each main meal each day for 60 days (A total of 3 capsules per day). The cinnamon capsules contained 600 mg of *Cinnamomum verum (Cinnamomum zeylanicum)* bark powder +100 mg of corn starch and placebo capsules contained 100 mg of corn starch, these capsules were produced with similar shape, color, and odor by Faculty of Pharmacy, Isfahan University of Medical Sciences. The dose was determined based on previous but the target group was a novelty in this study (Deyno et al., 2019). All patients were instructed to take their usual headache treatment medications because the adjuvant treatment alone is not ethical to administer. They were also requested to refrain from taking nonsteroidal anti-inflammatory drugs (NSAIDs) and not change their medication type and dose unless prescribed by their neurologist. Also, patients were encouraged to maintain their usual diet and routine physical activity throughout the intervention period.

**Socio-demographic and dietary intakes**

Demographic information was collected by a well-trained nutritionist. Using face-to-face interviews and a standard questionnaire, demographic data including age, sex, economic status, history of medications, family history of migraine, and baseline anthropometry status were taken from all participants. 

According to the current findings, dietary approaches, the glycemic index of the diet and the balance between the intake of essential fatty acids could be considered effective strategies in improving headache/ migraine (Razeghi Jahromi et al., 2019). Therefore, 3-day dietary records (2 week days and 1 week-end) at the beginning and the end of the trial were completed by all participants. Then, dietary intakes were entered into the Nutritionist IV software that was modified for Iranian foods.

**Headache daily result and disability assessment**

Headache daily result (HDR), the mean duration of migraine attacks per day (Miri et al., 2018), and the disability of migraine were determined by an experienced neurologist. Migraine disability was evaluated through a short and self-administered questionnaire that quantified headache-related disability (MIDAS questionnaire). This questionnaire had 5 questions; the total score is calculated based on the number of days marked against each question (Vasudha et al., 2018). The reliability and validity of the MIDAS questionnaire have been established in Iranian patients (Zandifar et al., 2014). Based on the total score, four grades were considered: Grade I with 0-5 score that means little or no disability; Grade II with 6-10 score that means mild disability; Grade III with 11-20 score that means moderate disability; and Grade IV with a score of more than 21 that means severe disability (Vasudha et al., 2018). 

**Anthropometric assessment**

Bodyweight (BW) in kilograms (kg) was measured by a calibrated digital scale with a measurement precision of 0.1 kg while wearing lightweight clothing and no shoes. The height (cm) was measured by a non-elastic tape and a measurement precision of 0.1 cm. BMI as the ratio between weight in kilograms and height in meters squared (kg/m^2^) was calculated. Waist circumference (WC) and hip circumference (HC), as a measure of aggregate fat, were assessed by a non-flexible tape to the nearest of 0.1 cm. WC was quantified in a standing position at the midpoint between the highest point of the iliac crest and lower part of the costal margin at the mid-axillary line. HC was evaluated from where the buttocks protrude the most. Waist-to-hip ratio (WHR) was calculated by dividing WC by HC.

**Statistical analyses**

The statistical analyses of data were carried out using the Statistical Package for the Social Sciences (SPSS) (Windows version 22.0, IBM Corp., Armonk, NY, USA). At first, data normality was determined by the Kolmogorov–Smirnov distribution test. Then, qualitative variables were compared by the chi-square test, which are expressed as frequencies (percentages). To compare the mean differences between the two groups at baseline, independent-sample t-test or Mann–Whitney test was performed. The paired-samples t-test or Wilcoxon test was used to check within group differences. Analysis of covariance (ANCOVA) was carried out to distinguish the effect of the intervention between the intervention and placebo groups, adjusted for age and sex. Data are reported as mean±SE. The test level for the statistical significance of differences in groups of the study was defined as p≤0.05.

## Results

**Figure 1:**
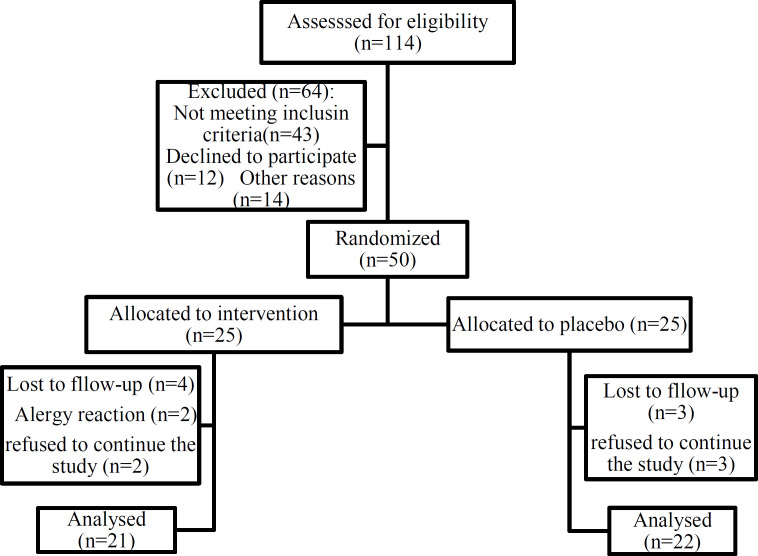
Chart of participants follow-up

From 114 patients who were diagnosed by one expert neurologist in the headache clinic, 50 participants met the eligibility criteria. During the study period (60 days), in the cinnamon group, two participants were excluded because of an allergic reaction including itching. Also, two participants in the cinnamon group and three participants in the placebo group refused to continue the study. Finally, 86% of the participants (21 in the intervention group and 22 in the placebo group) completed the study and were included in the final analysis (Figure 1). 

**Participant characteristics and nutrient assessment**

At baseline, no significant differences were observed between the two groups regarding anthropometric variables, age, gender, economic status, family history of migraine, or medications used (Table 1).

**Table 1:** Baseline demographic characteristics of migraine patients in the two groups of the study

**Variable**	**Cinnamon group (n=21)**	**Placebo (n=22)**	**p** **Value**
**Age (mean±SE (year))**	37.13±1.66	39.36±1.46	0.32^a^
**Gender (number (%))**			
**Female**	16 (76.19)	19 (86.36)	0.35^b^
**Male**	5 (23.80)	3 (13.63)	
**Family history of migraine (number (%))**	18 (85.71)	15 (68.18)	0.15^c^
**Weight (mean±SE (kg))**	70.02±2.42	70.50±3.46	0.64^a^
**Height (mean±SE (cm))**	164.13±2.27	161.45±1.60	0.34^ a^
**WC (mean±SE (cm))**	88.79±1.96	93.00±2.80	0.22^ a^
**HC (mean±SE (cm))**	105.47±1.33	101.06±4.63	0.36^ a^
**Economic status (number (%))**			
**Very low income**	5(23.80)	1 (4.54)	0.72^d^
**Low income**	7(33.33)	9 (40.90)	
**Average income**	8(38.09)	12 (54.54)	
**High income**	1(4.76)	0	
**Medications (number (%))**			
**Antidepressants**			
**Tricyclic Antidepressant**	2 (9.52)	4 (18.18)	0.31^b^
**SSRI**	3 (13.63)	3 (13.63)	1^b^
**SNRI**	2 (9.52)	2 (9.09)	1^b^
**Antiepileptic**	7 (33.33)	10 (45.45)	0.32^b^
**Gabapentin**	2 (9.52)	3 (13.63)	0.50^b^
**Beta-blockers**	5 (23.80)	8 (36.36)	0.32^b^

WC, Waist Circumference; HC, Hip Circumference; SSRI, Selective Serotonin Reuptake. Inhibitor; SNRI, Serotonin-Norepinephrine Reuptake Inhibitor. Notes: aIndependent samples t-test, bFisher exact test, cchi-square test, dMann–Whitney test.

**Table 2:** Dietary intake and physical activity of participants

**Variable**	**Cinnamon group (n=21)**				**Placebo group (n=22)**				**p** **Value**^b^
	**Baseline**	**After intervention**	**Mean Difference**	**p-Value** ^a^	**Baseline**	**After intervention**	**Mean Difference**	**p-Value** ^a^	
**Energy (Kcal)**	1854±168.27	1896±129.97	42.53±130.92	0.74	1919.71±96.43	1967.88±78.01	48.17±102.53	0.64	0.97
**Carbohydrate (g)**	287.53±37.69	281.05±38.74	-6.48±11.24	0.57	272.01±14.06	262.10±10.08	-9.90±13.28	0.46	0.85
**Glucose (g)**	6.68±0.91	7.36±1.21	0.67±1.55	0.66	8.44±0.89	6.98±0.78	-1.46±1.01	0.16	0.25
**Fructose (g)**	7.60±1.11	8.42±1.57	0.82±1.97	0.68	9.91±1.26	8.11±0.98	-1.79±1.29	0.18	0.26
**Galactose (g)**	0.82±0.25	0.88±0.22	0.06±0.27	0.80	0.44±0.08	0.64±0.19	0.19±0.20	0.36	0.71
**Lactose (g)**	5.98±1.13	5.00±1.27	-0.97±1.43	0.50	3.99±0.96	5.71±1.19	1.72±0.91	0.07	0.11
**Sucrose (g)**	10.72±2.29	12.03±2.27	1.30±1.51	0.39	12.99±2.73	11.19±2.17	-1.80±1.41	0.21	0.14
**Maltose (g)**	1.23±0.31	1.18±0.30	-0.04±0.36	0.89	1.58±0.35	1.25±0.27	-0.33±0.31	0.30	0.55
**Sugar (g)**	97.30±36.34	47.69±5.21	-49.60±34.22	0.16	54.31±5.14	48.16±4.93	-6.15±3.37	0.08	0.20
**Protein (g)**	62.24±4.86	68.07±5.32	5.82±4.77	0.23	67.40±5.15	71.00±5.55	3.85±2.73	0.17	0.72
**Fat (g)**	61.76±6.91	64.85±6.44	3.08±4.29	0.48	65.39±3.89	69.24±3.26	3.59±5.83	0.51	0.94
**Cholesterol (mg)**	218.41±35.98	238.50±24.29	20.09±39.92	0.62	199.77±22.35	231.15±22.66	31.38±32.24	0.34	0.82
**Saturated Fat (g)**	14.04±2.18	15.04±1.88	1.00±1.56	0.52	13.83±1.34	16.99±1.32	3.16±1.24	0.01	0.28
**Mono Fat (g)**	14.73±1.80	17.75±2.09	3.01±1.61	0.07	18.48±1.96	20.41±1.81	1.93±1.83	0.30	0.66
**Poly Fat (g)**	18.95±2.73	61.65±39.84	42.70±40.49	0.30	23.66±2.30	24.93±2.19	1.26±2.66	0.63	0.30
**Dietary Fibers (g)**	17.77±3.75	13.58±1.73	-4.19±2.23	0.07	15.15±0.89	14.93±0.91	-0.22±1.08	0.84	0.11
**Soluble Fiber (g)**	0.28±0.04	0.33±0.05	0.04±0.07	0.51	0.33±0.05	0.36±0.05	0.02±0.06	0.66	0.84
**Insoluble Fiber** **(g)**	1.38±0.26	1.59±0.23	0.21±0.34	0.54	1.30±0.19	1.75±0.23	0.45±0.26	0.09	0.57
**Crude Fiber (g)**	8.06±2.61	5.46±1.29	-2.59±1.46	0.09	5.92±0.38	5.57±0.36	-0.35±0.32	0.41	0.14
**Calcium (g)**	638.28±55.51	675.70±75.54	29.44±63.62	0.64	638.28±50.11	662.96±46.99	24.68±57.35	0.67	0.95
**Magnesium (g)**	227±28.14	231.85±30.46	3.96±13.61	0.77	231.28±12.22	238.52±14.46	7.24±11.42	0.53	0.85
**Vitamin D (mcg)**	0.66±0.18	0.65±0.24	-0.01±0.19	0.98	0.70±0.19	0.97±0.23	0.27±0.15	0.09	0.26
**Coffeine (g)**	74.31±10.15	79.19±8.27	4.87±7.82	0.54	66.70±9.39	79.39±7.69	12.68±8.06	0.13	0.49
**Physical activity** **(MET-min/week)**	565.93±119.64	638.22±223.28	72.29±169.72	0.42	616.59±183.2	683.07±211.87	66.47±136.40	0.61	0.63

Note: Data are reported as means±SE. a Paired t-test was used to compare pre-post tests. bIndependent T-Test was used to compare between the groups.

Also, in the 3-day dietary records, we considered total energy, macronutrients, and some micronutrients intake that are effective in losing weight. After data analysis, no significant differences between the groups were found in terms of dietary macro and micronutrients (Table 2).

**Change of** **headache daily result and disability**

After two months of intervention, HDR and disability significantly decreased in the cinnamon group in comparison to the placebo group (Table 3). The mean of HDR significantly reduced in both groups, however, the reduction was significantly greater in the intervention group compared with the placebo group (137.76±24.46 to 19.26±7.85 and 118.36±20.13 to 75.11±14.00 in the cinnamon group and placebo groups, respectively, p=0.006). The mean of total scores of disabilities remarkably reduced from 20.09±3.02 to 0.95±0.47 (p˂0.001) in the cinnamon group, while it did not significantly change in the placebo group (22.63±2.89 to 16.72±3.20, (p=0.54)). The difference between the two groups was statistically significant (p˂0.001). Likewise, the same results were found after adjusting for age and sex as shown in Table 3.

**Figure 2:**
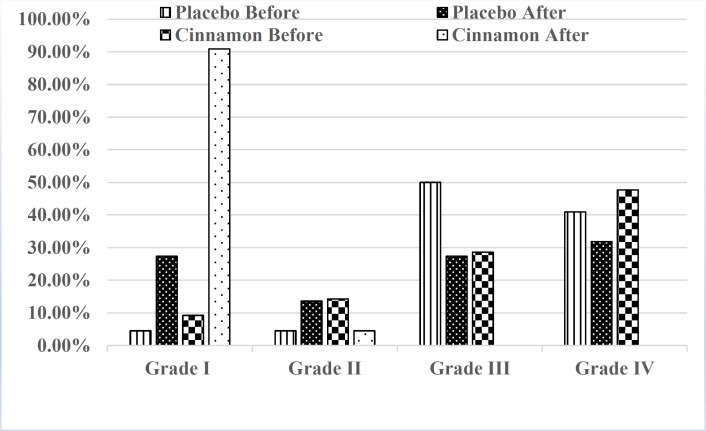
Effects of placebo and cinnamon on MIDAS grade in migraine patients

After intervention with cinnamon, the percentage of participants with Grades III and IV (moderate and severe disability) decreased from 76.18% to 0%, whereas those belonging to Grades I and II MIDAS (no or little and mild disability) enhanced from 23.8% to 95.4%. However, in the placebo group, participants with grades III and IV reduced from 90.90% to 59.10% and those belonging to Grades I and II MIDAS increased from 9.00% to 40.90%, respectively (Figure 2).

**Change of anthropometry measurements**

As shown in Table 4 after the intervention, BW and BMI did not change in the intervention group, however, both of them significantly increased in the placebo group. The differences between the two groups in BW and BMI were statistically significant (p<0.001). Compared with baseline, WC significantly decreased in the cinnamon group and remarkably increased in the placebo group; the difference between the groups was significant (p<0.001). Nevertheless, after the intervention, although HC significantly decreased in the intervention group, no significant difference was found between the groups in the crude model. Likewise, WHR (WC/HC) did not significantly change in the intervention compared with the placebo group in the crude model. However, after adjustment for age and sex, the ANCOVA test showed that reduction in HC and WHR in the intervention group was significant compared with the placebo group (p=0.001 and p=0.008, respectively) (Table 4).

**Table 3:** Change in disability and headache daily in migraine patients after 2 months of intervention

**Parameter**		**Cinnamon group (n=21)**	**Placebo (n=22)**	**p-Value** ^b^	**p-Value** ^c^
**MIDAS Score (Day)**	Before	20.09±3.02	22.63±2.89		
	After	0.95±0.47	16.72±3.20		
	Differences	-20.09±3.12	-5.90±2.20		
	p Value^a^	˂0.001	0.54		
	Between-Group Difference			˂0.001	˂0.001
**HDR**	Before	137.76±24.46	118.36±20.13		
	After	19.26±7.85	75.11±14.00		
	Differences	118.50±22.09	43.25±14.19		
	p Value^a^	˂0.001	0.006		
	Between-Group Difference			0.006	˂0.001

MIDAS, Migraine Disability Assessment Questionnaire; HDR, Headache Daily Result. Note: Data are reported as means±SE. ^a^Paired t-test was used to compare pre-post tests. ^b^Independent samples t-test was used to compare between groups. ^c^ANCOVA test (adjusted for age and sex)

**Table 4:** Anthropometric measurement in migraine patients before and after the intervention

**Variable**		**Cinnamon group (n=21)**	**Placebo (n=22)**	**p Value** ^b^	**p Value** ^c^
**Weight (kg)**	Before	70.02±2.42	70.50±3.46		
	After	70.09±2.57	72.15±3.54		
	Differences	-0.17±0.33	1.64±0.33		
	*P *Value^a^	0.60	˂0.001		
	Between-Group Difference			˂0.001	0.001
**BMI (kg/m** ^2^ **)**	Before	26.12±3.35	26.87±4.77		
	After	26.06±3.51	27.50±4.86		
	Differences	0.64±0.57	-0.62±0.60		
	*P *Value^a^	0.61	˂0.001		
	Between-Group Difference			˂0.001	0.001
**WC (cm)**	Before	88.79±1.96	93.00±2.80		
	After	88.10±1.96	94.25±2.86		
	Differences	-1.35±0.47	1.25±0.38		
	*P *Value^a^	0.01	0.004		
	Between-Group Difference			˂0.001	˂0.001
**HC (cm)**	Before	105.47±1.33	101.06±4.63		
	After	104.92±1.35	106.07±1.62		
	Differences	-0.80±0.27	5.00±4.09		
	*P *Value^a^	0.008	0.23		
	Between-Group Difference			0.17	0.001
**WHR**	Before	0.84±0.06	0.88±0.08		
	After	0.83±0.06	0.88±0.08		
	Differences	-0.006±0.01	0.003±0.01		
	*P *Value^a^	0.10	0.33		
	Between-Group Difference			0.06	0.008

BMI, Body Mass Index; WC, Waist Circumference; HC, Hip Circumference; WHR, Waist-to-Hip Ratio. Note: Data are reported as means±SE. ^a ^Paired t-test was used to compare pre-post tests. ^ b^Independent samples t-test was used to compare between groups. ^c^ANCOVA test (adjusted for age and sex)

## Discussion

The principal finding of the current study was that *Cinnamomum verum* powder intake significantly prevented weight gain, increasing BMI and WC, and reduced HC and WHR in migraine patients compared with the placebo group. It also significantly reduced the rate of disability in patients by decreasing the HDR in moderate to severe grades. With regard to the bilateral relationship between obesity and migraine frequency, our findings might be useful in clinical settings to prevent obesity and the development of migraine headaches thus improving the functional capacity of migraine patients.

Although the mechanisms contributing to migraine pathophysiology are not completely clear, recent evidence has indicated that the neuroinflammatory state has a critical role in the development of migraine attacks (Longoni and Ferrarese, 2006). One of the factors that cause inflammation in the body is the increase in body fat (Ford et al., 2008). Also, there has been some evidence that indicates that obesity is comorbid with increasing the severity and frequency of migraine attacks (Huang and Liang, 2018). 

The weight gain leads to the expansion of adipose tissue and creates a state of chronic low-grade inflammation. Also, the adipose tissue, as a neuroendocrine organ has a role in energy homeostasis and inflammation by adipokines including cytokines, such as tumor necrosis factor TNF-α, interleukins (IL-1, IL-6, and IL-10), leptin, resistin, adiponectin, chemokines, such as IL-8, etc. These adipocytokines are one of the suggested causes of the association between obesity and migraine (Di Renzo et al., 2018). Most patients suffering from migraines have to use the medications (Silberstein, 2015). One side effect of some common drugs used to treat migraines is weight gain which occurs as a result of increased appetite. Therefore, one of the main challenges regarding migraine medication is weight gain and an increase in BMI of patients (Whyte and Tepper, 2009; Young, 2008). 

Considering several challenges to follow a healthy lifestyle, particularly for a long time for some of the patients, herbal supplements without considerable side effects are an attractive alternative to prevent the side effects of medications such as overweight and obesity (Wells et al., 2017). In the current study, we found that cinnamon could be effective to prevent weight gain among the migraine patients, which is in accordance with the results of the previous reviews which showed that cinnamon is an effective agent for weight loss and improving metabolic syndrome (Mollazadeh and Hosseinzadeh, 2016; Mousavi et al., 2020). 

To explain these results, it should be mentioned that cinnamon is a potent antioxidant and anti-obesity herb because of its high amounts of polyphenols, flavanols, and cinnamaldehyde (Farhat et al., 2017; Mollazadeh and Hosseinzadeh, 2016). Cinnamon lessens glucose absorption in the small intestine by postponing gastric emptying, increasing glucosidase enzymes, and inhibiting ATPase of intestinal brush borders. In addition, activating glycogen synthase and inhibiting glycogen synthase kinase 3β lead to decrease glycogenolysis, and increase glycogen synthesis (Hafizur et al., 2015). Also, polyphenolic compounds of cinnamon with anti-obesogenic effects can inhibit lipolysis, lipogenesis, and intestinal lipid absorption (Mercader et al., 2011). Also, these induce fatty acid oxidation and antagonism at cannabinoid receptors (Seely et al., 2009). Therefore, these actions can be effective in decreasing the synthesis and storage of fat and improvement of anthropometric status.

Another compound in cinnamon, which might be effective against increasing weight is Methyl Hydroxy chalcone polymers (MHCP). MHCP causes the enhancement of insulin sensitivity in adipose cells and helps the increasing body metabolism through activating the insulin-receptor kinase and inhibiting the insulin-receptor-phosphatase (Mousavi et al., 2020).

Cinnamon consumption can prevent proinflammatory cytokines production, oxidative stress condition, and the risk of developing a number of chronic diseases, including cancer, heart disease, and diabetes caused by insulin resistance in obese migraine patients (Mollazadeh and Hosseinzadeh, 2016). Also, it was shown that cinnamon is effective to reduce inflammatory factors IL-6 and nitric oxide in people with migraine (Zareie et al., 2020). It is proposed that cinnamaldehyde, as the main bioactive component of cinnamon, has a salient role in reduction of production of inflammatory cytokines by suppressing the expression of cyclooxygenase and nitric oxide synthase (Zareie et al., 2020). Generally, cinnamon's components and metabolites have modulatory effects on the release of inflammatory mediators (Modi et al., 2015).

In addition to preventing weight gain, in the current study, cinnamon reduced disability in moderate to severe sufferers of migraines by reducing the HDR. Also, according to the evidence, reduction in disability of migraine patients may result in increasing the productivity of individuals in work, school, homework, and generally social or leisure activities. Therefore, cinnamon powder consumption might be a novel approach to reduce the headache disability and control the weight gain in such a population. It seems that cinnamon with anti-inflammatory properties reduces the production of inflammatory cytokines (Ho et al., 2013) and headache attack time.

Although this study was the first randomized double-blind clinical trial that investigated the effect of cinnamon as a natural, inexpensive, and accessible herbal medicine on anthropometry factors and headache disability in migraine, some limitations should be noted. The duration of the intervention was relatively short and there was no long-term follow-up. More randomized controlled trails are warranted before causal relationship can be established. Moreover, because of ethical issues, the dosage of cinnamon was considered with caution. Therefore, longer and larger trials with different dosages of cinnamon are recommended.

The results revealed that cinnamon consumption was able to control the weight gain and reduced waist and hip circumference in patients with migraine after results were adjusted for age and sex. Furthermore, the headache disability and headache daily result significantly decreased in these patients. Our findings implied that cinnamon might be considered a complementary medicine in migraine patients, though further studies are needed to confirm our results.

## Conflicts of interest

The authors have declared that there is no conflict of interest.
